# Extracellular matrix derived from human urine-derived stem cells enhances the expansion, adhesion, spreading, and differentiation of human periodontal ligament stem cells

**DOI:** 10.1186/s13287-019-1483-7

**Published:** 2019-12-18

**Authors:** Xue Xiong, Xiao Yang, Hongwei Dai, Gang Feng, Yuanyuan Zhang, Jianping Zhou, Wenwen Zhou

**Affiliations:** 1 Department of Orthodontics, Stomatological Hospital of Chongqing Medical University, No. 426, North Songshi Road, Yubei District, Chongqing, 401147 China; 2 Chongqing Key Laboratory of Oral Diseases and Biomedical Sciences, Chongqing, China; 3 Chongqing Municipal Key Laboratory of Oral Biomedical Engineering of Higher Education, Chongqing, China; 4 The Institute for Regenerative Medicine, School of Medicine, Wake Forest University, Winston-Salem, NC USA

**Keywords:** Extracellular matrix, Human urine-derived stem cells, Human periodontal ligament stem cells, Tissue culture plastic, Fibronectin, Cell expansion, Cell adhesion, Osteogenesis, Angiogenesis, Adipogenesis

## Abstract

**Background:**

Human periodontal ligament stem cells (hPDLSCs) are one of the most promising types of seed cells in periodontal tissue regeneration. Suitable biomaterials are additional essential components that must cooperate with seed cells for in vivo expansion or in vitro implantation. Extracellular matrix (ECM) derived from mesenchymal stem cells (MSCs) was recently reported to be a promising substrate with which to culture MSCs that could be applied in biomaterial scaffolds or bioink. Human urine-derived stem cells (hUSCs) have several advantages; their collection is non-invasive and easy, and hUSCs are low in cost, potentially making them a suitable and efficient source of ECM. The purpose of this study was to characterize the biological properties of ECM derived from hUSCs (UECM) and evaluate the effects of UECM on hPDLSCs.

**Methods:**

hPDLSCs grown on ECM derived from hPDLSCs (PECM) and fibronectin-coated tissue culture plastic (TCP) served as control groups. Both hUSCs and hPDLSCs were seeded on TCP and stimulated to produce ECM. After 8 days of stimulation, the samples were decellularized, leaving only ECM. Then, hPDLSCs were seeded onto UECM-, PECM-, and fibronectin-coated TCP and untreated TCP.

**Results:**

UECM consists of dense bundles of fibers which contain abundant fibronectin. Both UECM and PECM promoted hPDLSC proliferation, attachment, spreading, and differentiation. Between UECM and PECM, UECM enhanced proliferation, osteogenesis, and angiogenesis to a greater extent. Though fibronectin appeared to be the abundant component of UECM, its performance was inferior to that of UECM.

**Conclusions:**

Our study provides an original perspective on different cell-specific ECMs and suggests UECM as a suitable biomaterial in which to culture hPDLSCs as UECM enhances their biological functions.

## Background

A large proportion of the worldwide population suffers from the destruction of periodontal tissues due to periodontal disease, trauma, and tumors. Periodontal disease, including gingivitis and periodontitis, affects up to 90% of people worldwide [1]. Gingivitis will not cause alveolar bone destruction while the advanced forms of periodontitis affect 10–15% of the population worldwide leading to severe periodontal tissue destruction and tooth loss [2]. Anti-infective and mechanical therapies for periodontal disease mainly work by eliminating inflammation and preventing progressive bone loss but cannot induce periodontal tissue regeneration. Other regenerative therapies, such as guided tissue regeneration (GTR), enamel matrix derivatives (EMDs), and platelet-rich plasma (PRP), have achieved some success, but knowledge of the prognoses of these therapies is limited and unclear [3–6]. Mesenchymal stem cells (MSCs), which undergo self-renewal and have multilineage potential, can renew themselves for a long time without significant changes in phenotype and function. MSCs are able to differentiate into other cell lineages, such as bone, fat, cartilage, and muscle, making cell therapy-based tissue regeneration possible [7, 8]. Seo et al. found periodontal ligament stem cells in 2004. This kind of tissue-specific stem cell line can differentiate into cementoblast/osteoblasts and adipocytes under certain culture conditions and is therefore a promising choice for use in periodontal tissue regeneration [9]. A series of studies have demonstrated that bone marrow stromal cells (BMSCs), alveolar periosteal cells (APCs), and periodontal ligament cells (PDLCs) can be used as seed cells, with good effects observed in large animal models. Yuka et al. compared three types of cells (BMSCs, APCs, PDLCs) and found that PDLCs were the most suitable cell source for complex periodontal tissue [3, 10].

However, to meet the needs of cell therapies that require the large-scale expansion of MSCs remains challenging. The biological characteristics of MSCs were altered when they were cultured on tissue culture plastic (TCP) or adsorbed protein (such as fibronectin or collagen) during long-term in vitro culture [11, 12]. To solve this problem, some researchers cultured hBMSCs on self-derived decellularized extracellular matrix (ECM), showing that autologous ECM can enhance ex vivo expansion, attachment, spreading, and differentiation potential while maintaining MSC stemness [12–18]. These results illustrate that ECM derived from MSCs is a promising substrate in which to culture stem cells.

All cells are pertinent to ECM, which plays a crucial role in regulating stem cell fate in particular. Different types of ECM vary in composition and function [19]. ECM can provide mechanical support and enhance the mobility and adhesion of cells. Furthermore, when cells bind to ECM, ECM can regulate intracellular signaling via cell surface adhesion receptors, of which the integrin family is the principle and major member [19–22]. Additionally, multiple protein domains in the ECM can bind and release corresponding growth factors in a sustained fashion so that ECM can synergize with growth factors to affect cellular behavior [22]. Moreover, the different micropatterned islands, stiffness, physical topography, and deformability of ECMs can promote different biological processes [19].

In periodontal regeneration engineering, Wang et al. cultured hPDLSCs on ECM membranes isolated from porcine urinary bladders and demonstrated that the ECM membrane enhanced their proliferation and regenerative capabilities [23]. Tissue-derived ECM has the advantage of being easy to harvest and can increase the expansion of MSCs. However, it is animal-derived and expensive, and different batches exhibit variations [12, 23]. Adsorbed monolayers of single ECM components, such as fibronectin, collagen I, and laminin, isolated artificially fail to mimic the complex structure and complete function of natural ECM [12, 24]. ECM derived from MSCs promotes the proliferation, adhesion, spreading, and differentiation potential of stem cells to a greater degree than other kinds of ECM described above, including ECM produced by non-stem cells, and it can rejuvenate aged stem cells without the loss of their characteristics [12, 14–16, 18]. Nonetheless, many stem cells must be sacrificed to produce ECM, making mass production difficult. The present research mainly focuses on the ECM of hBMSCs obtained by invasive operations, which is limited by its source and quantity [12, 15, 16, 18]. Most human stem cells require invasive and complex procedures, leading to complications that include bleeding, infection, and excess pain. A new solution to this problem was suggested in 2008 by Zhang et al., who isolated stem cells from human urine and named them human urine-derived stem cells (hUSCs) [25].

hUSCs, which have the characteristics of MSCs, including clonogenicity, self-renewal, and multidifferentiation potential, have the advantages of being non-invasive, easy to collect, and low in cost, making them a suitable and efficient source of ECM [25–27]. Pei et al. cultured hBMSCs on ECM deposited by hUSCs (UECM) and showed that UECM strengthened the chondrogenic capacity of repeated passages of hBMSCs through the Wnt11-mediated non-canonical signaling pathway [28].

Because few studies have investigated the influence of native or foreign ECM on hPDLSCs, this study was conducted to examine whether UECM is a suitable and efficient substrate in which to culture hPDLSCs in vitro and in vivo. To assess interactions between hPDLSCs and their native ECM, we examined one group containing ECM deposited by hPDLSCs (PECM) and compared the influences of UECM and PECM on hPDLSCs. TCP coated by fibronectin, the adsorbed monolayers of single ECM components, also served as a control group.

## Materials and methods

### Sample collection and cell culture

Human USCs and PDLSCs were both isolated and cultured according to slightly modified previous protocols [9, 25].

#### USC culture

Two hundred milliliters of fresh midstream urine was collected from 8 healthy male volunteers (20–25 years old). Every 50 ml of sample was centrifuged at 500×*g* for 5 min at room temperature. After the supernatants were discarded, the sedimented cells were washed with phosphate-buffered saline (PBS, Sigma-Aldrich, USA). Then, the sediments were resuspended in keratinocyte serum-free medium (K-sfm, Gibco BRL, USA) and progenitor cell medium in a 1:1 ratio. K-sfm contained 50 ng/ml bovine pituitary extract (Science Cell, USA), 5 ng/ml epidermal growth factor (Sigma-Aldrich), 30 ng/ml cholera toxin (Sigma-Aldrich), and 1 mg/ml streptomycin (HyClone, USA). Progenitor cell medium was composed of 75% Dulbecco’s modified Eagle’s medium (DMED, HyClone), 25% Nutrient Mixture F-12 Ham (Gibco BRL), 10% fetal bovine serum (FBS, Gibco BRL), 10 ng/ml epidermal growth factor (Sigma-Aldrich), 0.4 μg/ml hydrocortisone (Sigma-Aldrich), 5 ng/ml insulin (Sigma-Aldrich), 5 μg/ml transferrin (Sigma-Aldrich), 2 × 10^−9^ M3,3′,5-triiodo-l-thyronine (Gibco BRL), 1.8 × 10^−4^ M adenine (Sigma-Aldrich), 10^−10^ M cholera toxin (Sigma-Aldrich), and 1% penicillin-streptomycin (Gibco BRL). After resuspending, the obtained cells were seeded into 24-well culture plates and incubated at 37 °C in a humidified atmosphere with 5% CO_2_. The medium was changed every 2 or 3 days. Cells were passaged when they reached approximately 80% confluence.

#### PDLSC culture

Healthy premolars extracted for orthodontic treatment were collected from donors 12 to 18 years of age. The periodontal ligament tissue was gently separated from the middle 1/3 of the root surface and minced into approximately 1.0 mm^3^ fragments. The fragments were subsequently digested with 3 mg/ml collagenase type 1 (COL-1, Sigma-Aldrich) for 30 min in a water bath at 37 °C. The digested solution was then centrifuged at 500×*g* for 10 min. After centrifugation, the digested tissue was tiled on the bottom of T25 culture bottles (Corning, USA) with 5 ml α-minimum essential medium (α-MEM, HyClone) supplemented with 10% FBS (Gibco BRL) and 1% penicillin-streptomycin (Gibco BRL) and incubated in 5% CO_2_ at 37 °C. The culture bottles were inverted overnight and turned over the second day. The medium was changed every 2 or 3 days. The cells were passaged when they reached approximately 80% confluence.

### Characterization of USCs and PDLSCs

#### Flow cytometric analysis of cell phenotype

Cell surface markers in hPDLSCs (P3) and USCs (P3) were examined by flow cytometric analysis.

Approximately 1 × 10^6^ hPDLSCs were incubated for 1 h in the dark with the FITC-conjugated monoclonal antibodies against the following: CD31 (Becton, Dickinson and Company, USA), CD34 (BD), CD45 (BD), CD90 (BD), CD105 (BD), and CD146 (BD). Cell suspensions incubated with isotype control antibodies were used as controls. Cells were washed twice to remove non-specific binding and then analyzed with a BD Influx flow cytometry system.

#### Osteogenic/adipogenic differentiation

##### Osteogenic induction

P3 PDLSCs and USCs were seeded at a density of 1 × 10^4^ cells/cm^2^ in six-well plates (Corning, USA). When they reached 80% confluence, the general growth medium was changed to osteogenic medium (α-MEM containing 10% FBS, 10 mM β-glycerophosphate (Sigma-Aldrich), 10 nM dexamethasone (Sigma-Aldrich), 50 μg/ml ascorbic acid (Sigma-Aldrich), and 1% penicillin-streptomycin). After osteogenic induction for 21 days, alizarin red (alizarin red S; Beyotime, China) was utilized to detect mineral deposit formation.

##### Adipogenic induction

Cultured hUSCs and hPDLSCs were grown to 100% confluence and then incubated with adipogenic medium (DMEM containing 10% FBS, 1 μM dexamethasone, 100 μM indomethacin, 0.5 mM 3-isobutyl-1-methylxanthine (Sigma-Aldrich), 10 μM insulin, and 1% penicillin-streptomycin). After 21 days, the formation of lipid-containing areas was determined by Oil Red O staining (Solarbio, China).

### Preparation of decellularized ECM from USCs and PDLSCs and fibronectin-coated substrates

P4 hUSCs and hPDLSCs were seeded at a density of 2 × 10^4^/cm^2^. When cells on TCP had grown to 80–90% confluence, the medium was changed to supplemented medium (SM: α-MEM with 10% FBS, 50 μg/ml l-ascorbic acid, and 1% penicillin-streptomycin). Cells were continually cultured in SM for 8 days, and the medium was changed every 2 or 3 days. The cells were rinsed with PBS and treated with 0.5% Triton X-100 (Sigma, St. Louis, MO) containing 20 mM NH_4_OH in PBS for 5 min at 37 °C. To obtain cell-free ECM, the plates were rinsed with PBS twice and subsequently treated with 100 U/ml DNase (Sigma-Aldrich) at 37 °C for 1 h to remove remaining DNA. After washing with PBS three times, ECMs from USCs (UECM) and PDLSCs (PECM) were allowed to dry in a sterile biosafety cabinet. Then, they were insulated in a sterile container and stored at 4 °C in the dark for up to 1 month before use. Fibronectin-coated substrates were prepared according to the manufacturer’s instructions. Briefly, 2.5 μg/cm^2^ fibronectin (Sigma-Aldrich) in PBS was absorbed to tissue culture substrates by incubating for 2 h at room temperature. After rinsing with PBS, the plates were used for cell culture.

### Analysis of UECM and PECM

#### Scanning electronic microscopy

hUSC- and hPDLSC-secreted ECMs on plastic overlaps were washed three times with PBS, fixed in 2.5% glutaraldehyde for 2 h, and then fixed in 2% osmium tetroxide. ECMs were dehydrated using an ethanol gradient (25%, 50%, 75%, 95%, and 100%) for 10 min for each concentration, followed by infiltration with hexamethyldisilazane (HMDS) and ethanol at a ratio of 1:1 twice for 1 h each time and incubation in HMDS and ethanol at a ratio of 1:2 overnight, ending with 3 incubations in 100% HMDS for 4 h each. Samples were coated with gold sputter, and images of the ECM were taken using a scanning electron microscope (Hitachi S-4800, Japan).

#### Immunofluorescent staining

UECM samples on plastic slides were fixed with 4% paraformaldehyde in PBS for 30 min. After incubation in 10% normal goat serum for 30 min at room temperature, the samples were treated with primary antibodies against type I collagen (Abcam, UK), fibronectin (Abcam), and laminin 5 (Abcam) at 4 °C overnight, followed by incubation in Alexa Fluor 488-conjugated goat anti-rabbit IgG (Bioss, China) and Alexa Fluor 647-conjugated goat anti-mouse IgG (Bioss) for 30 min. Images were recorded by confocal laser scanning microscopy (Leica TCS SP8 X, Germany).

### Cell proliferation

PDLSCs were seeded a density of 3000/cm^2^ in uncoated 24-well plates or 24-well plates coated with UECM, PECM, and fibronectin. At days 1, 3, 5, 7, and 9 after cell seeding, cell proliferation was evaluated with a CCK-8 assay (CCK-8 kit, Dojindo, Japan) following the manufacturer’s instructions.

### The dynamics of cellular adhesion: static and dynamic methods

hPDLSCs adhesion on different ECM substrates was measured by both static and dynamic methods according to the methods of a previous study [15, 29]. These analyses were performed as follows: (1) Static method—PDLSCs were seeded at a density of 50,000/cm^2^ in 2 ml serum-free cell culture medium on untreated 12-well plates and 12-well plates coated with ECMs from different sources. After 1 h, the supernatant was collected, and non-adherent cells were counted. (2) Dynamic method—This procedure was similar to the static method, except the cell-seeded plates were placed on a shaking platform at 80 rpm for 1 h during the incubation period.

### Spreading and morphology of cells grown on ECM

hPDLSCs were seeded at a density of 10^3^/cm^2^ on laser confocal dishes coated with different substrates. After 6 h of cultivation, the non-adherent cells were washed away, and the samples were fixed with 4% paraformaldehyde for 10 min at room temperature. The samples were then permeabilized with 0.2% Triton X-100 for 5 min. Following 3 rinses with PBS on ice, the cells were stained with Alexa Fluor 555-labeled phalloidin (Yeasen, China) and DAPI (Bioss). Images were recorded by confocal laser scanning microscopy (Leica TCS SP8 X). The cell spreading area and the average optical density were analyzed using ImageJ (Additional file 1, Additional file 2, Additional file 3, and Additional file 4).

### hPDLSC differentiation on ECM-coated surfaces

hPDLSCs were seeded onto non-coated and ECM-coated wells and cultured in osteogenic, adipogenic, and angiogenic medium (endothelial growth medium 2, (EGM-2; Lonza Biologics, USA) with 2% FBS and 50 ng/ml vascular endothelial growth factor (VEGF, Sino Biological, China)). After 7 days of culture, RNA was extracted from the cultures using RNAiso (TaKaRa, Japan) and qualified spectrophotometrically (A260/A280) with a Nanodrop spectrophotometer (Thermo Fisher Scientific, USA). Then, RNA was reverse transcribed into cDNA using RT Master Mix (TaKaRa). Real-time polymerase chain reaction (RT-PCR) was performed with SYBR Green detection reagent (SYBR Premix, TaKaRa) to analyze gene expression. The expression of genes related to osteogenic differentiation (alkaline phosphatase (ALP), RUNX2, osteocalcin (OCN), periostin (POSTN)), adipogenic differentiation (LPL, PPARγ2), and angiogenic differentiation (VEGF-A, CD31) were analyzed in hPDLSCs cultured in 3 different types of induction media on UECM, PECM, fibronectin, and TCP. Glyceraldehyde 3-phosphate dehydrogenase (GAPDH) was used as a housekeeping gene. The sequences of the primers used to amplify these genes were as follows: GAPDH—TTGGTATCGTGGAAGG and ACAACTGCCATCACGCCACAGTTTC, ALP—TGGCAGTGTCCAGGGAAGAA and AACGCAGGATTTCCCACACTA, RUNX2—TCAACGATCTGAGATTTGTGGG and GGGGAGGATTTGTGAAGACGG, OCN—CCCAGGCGCTACCTGTATCAA and GGTCAGCCAACTCGTCACAGTC, POSTN—ACTTTGCTGGCACCTGTGAATA and TCCGATGGTTTCCAGTATTTGC, LPL—CCAGAAACCAGTTGGGCATGT and GCTGGTCCACATCTCCAAGTCC, PPARγ2—GGCCGAGAAGGAGAAGCTGT and ACCTGGGCGGTTGATTTGTC, VEGF-A—GAGG- GCAGAATCATCACGAAGT and GCACACAGGATGGCTTGAAGA, and CD31—CACCAAGATAGCCTCAAAGTCGG and GGCTGGGAGAGCATTTCACATAC.

Western blotting was conducted on day 7 to further detect the osteogenic differentiation potential of hPDLSCs. Total protein was extracted using RIPA Lysis Buffer (Beyotime) on ice with a protease inhibitor cocktail (Beyotime). The protein concentrations were quantified using a BCA Protein Assay Kit (Beyotime). Fifty micrograms of protein from each sample were separated by sodium dodecyl sulfate-polyacrylamide gel electrophoresis (SDS-PAGE) and then transferred to polyvinylidene fluoride (PVDF) membranes (GE Healthcare, UK). The membranes were blocked with 5% non-fat dry milk dissolved in TBS-Tween 20 for 1 h and subsequently incubated overnight at 4 °C with primary antibodies against the following: RUNX2 (Abcam), POSTN (Abcam), and GAPDH (Bioss), which served as an internal control, followed by incubation with HRP-conjugated secondary antibodies (goat anti-rabbit IgG (Bioss)) for 1 h. Targeted proteins were detected with an ECL Plus Western Blotting Detection System (GE Healthcare, UK).

After induction for 21 days, alizarin red staining and Oil Red O staining were performed following the manufacturer’s instructions to detect mineral deposits and lipid vesicles.

### Flow cytometric analysis of hPDLSCs on ECM-coated surfaces

P4 hPDLSCs were cultured on 4 different surfaces for 1 passage in general medium. Then cells were digested, and cell surface markers were assessed as described above.

### In vivo studies

In vivo studies were carried out based on the methods of previous studies [18, 30]. First, UECM and PECM were thoroughly mixed with hydroxyapatite (HA) powder (Zimmer, USA) using an injection syringe. At first, hPDLSCs were respectively cultured on UECM, PECM, and untreated TCP in osteogenic media for 7 days. Then, hPDLSCs cultured on TCP were digested and mixed with untreated HA powder (HA group) while hPDLSCs cultured on UECM were mixed with prepared HA powder mixed with UECM (HA/UECM group) and hPDLSCs cultured on PECM were mixed with prepared HA powder mixed with PECM (HA/PECM group). Next, the mixtures were implanted subcutaneously into the bilateral dorsal surfaces of nude mice (4- to 6-week-old males; Chongqing Medical University Animal Center, Chongqing, China) (6 animals per testing group). After 6 weeks, the implants were harvested, fixed in 4% paraformaldehyde, decalcified with 5% EDTA (pH 8.0) at room temperature for 2 weeks, and embedded in paraffin. To track osteogenic differentiation of the implanted PDLSCs, slides of grafted tissues (5 μm) were assessed by H&E staining, Masson staining, and immunohistochemical staining using antibodies against human POSTN (Abcam) and OCN (Abcam). Histologic slides were visualized under a Leica DM4000B fluorescence microscope, and images were recorded for analysis.

### Statistical analysis

Data are presented as the mean ± SD. Statistical analysis was performed with one-way analysis of variance (ANOVA), followed by Tukey’s post hoc multiple comparisons test (GraphPad Prism 8). Differences were considered significant when *p* < 0.05 (*) and *p* < 0.01 (**).

## Results

### Characterization of hUSCs and hPDLSCs

**Fig. 1:**
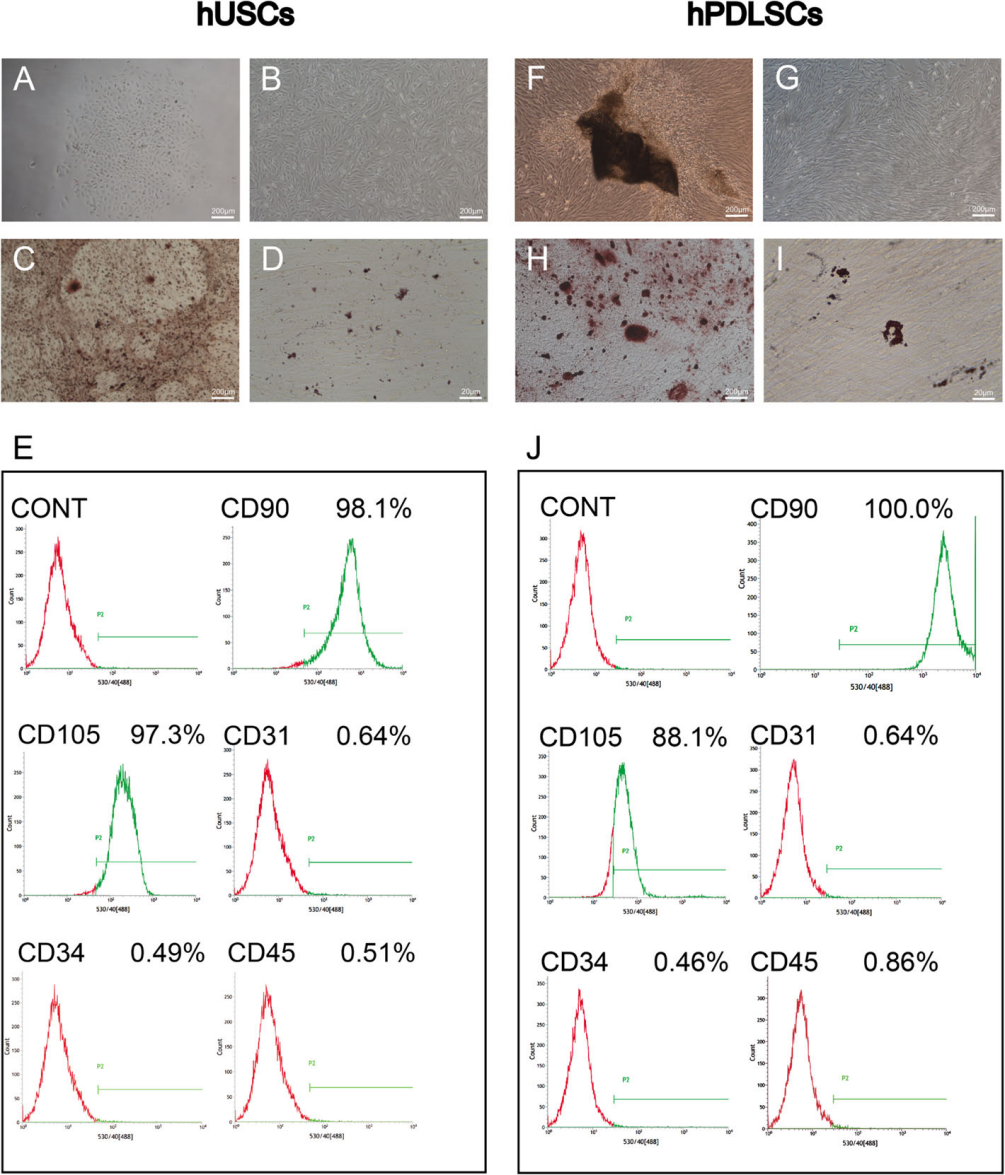
Characterization of hUSCs and hPDLSCs. **a**, **f** Primary hUSCs and hPDLSCs. **b**, **g** hUSCs and hPDLSCs grown in culture medium and exhibiting rice grain-like and long spindle-shaped morphology, respectively. **c**, **h** After osteogenic induction for 21 days, mineral deposits formed by hUSCs and hPDLSCs were stained with alizarin red. **d**, **i** Cultured in adipogenic medium for 21 days, lipid-laden lobules formed by hUSCs and hPDLSCs were detected with Oil Red O. **e**, **j** Cytometric flow analysis indicated that both hUSCs and hPDLSCs expressed the mesenchymal associated markers CD90 and CD105, and were negative for CD31, CD34, and CD45

After 5–8 days, hUSCs and hPDLSCs were both observed by inverted phase-contrast light microscopy (Fig. 1a). hUSCs showed a rice grain-like morphology, while hPDLSCs showed a spindle-shaped morphology (Fig. 1b). To determine whether hUSCs and hPDLSCs have the potential to differentiate into multiple lineages, they were cultured in osteogenic and adipogenic media for 21 days. Then, alizarin red and Oil Red O staining were performed. Calcium mineral deposits and lipid-laden lobules were both observed in both hUSCs and hPDLSCs, which indicated the differentiation potential of hUSCs and hPDLSCs (Fig. 1c, d, h, i). Both P3 hPDLSCs and P3 hUSCs were positive for CD90 and CD146 and negative for CD31, CD34, and CD45 (Fig. 1e, j). This surface phenotype is similar to that of MSCs.

### Characteristics of hUSC- and hPDLSC-derived ECM

**Fig. 2:**
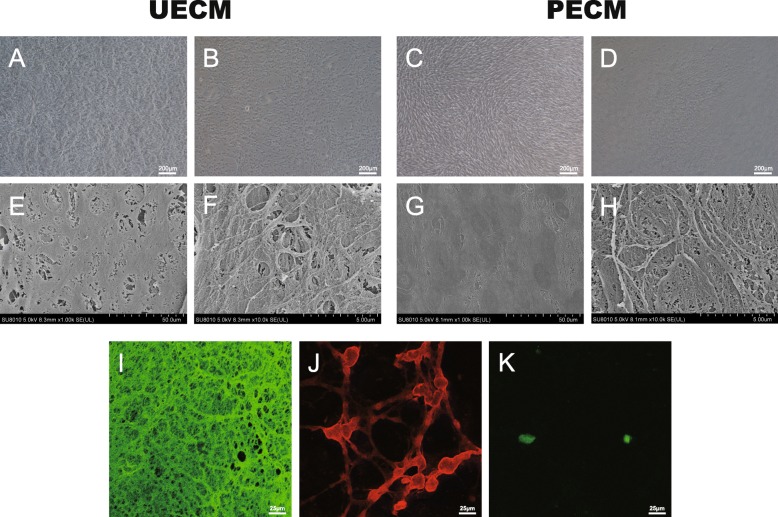
Characterization of UECM and PECM. **a**, **b** Images of hUSCs and **e**, **f** hPDLSCs before and after decellularization obtained with a phase contrast microscope. Scale bar = 200 μm. **c**, **d** SEM images of UECM at × 1000 and × 10,000 magnification. **g**, **h** SEM images of PECM at × 1000 and × 10,000 magnification. **i**–**k** Immunostaining for the presence of ECM proteins. **i** Fibronectin. **j** Collagen I. **k** Laminin 5. Scale bar = 25 μm

hUSCs and hPDLSCs were observed by inverted phase-contrast light microscopy before decellularized when cultured in SM for 8 days (Fig. 2e, a, c). After removal of the cells, ECM secreted by hUSCs and hPDLSCs was observed by inverted phase-contrast light microscopy and SEM, which revealed that UECM and PECM are both composed of dense bundles of fibers with small spaces between the fibers (Fig. 2e–h). Immunofluorescent staining revealed that the fibronectin is more abundant than collagen I and laminin. Laminin was sparser and far less abundant than the other two components observed (Fig. 2i–k). These results are similar to those reported by Pei et al. [28].

### hPDLSC proliferation on ECM

**Fig. 3:**
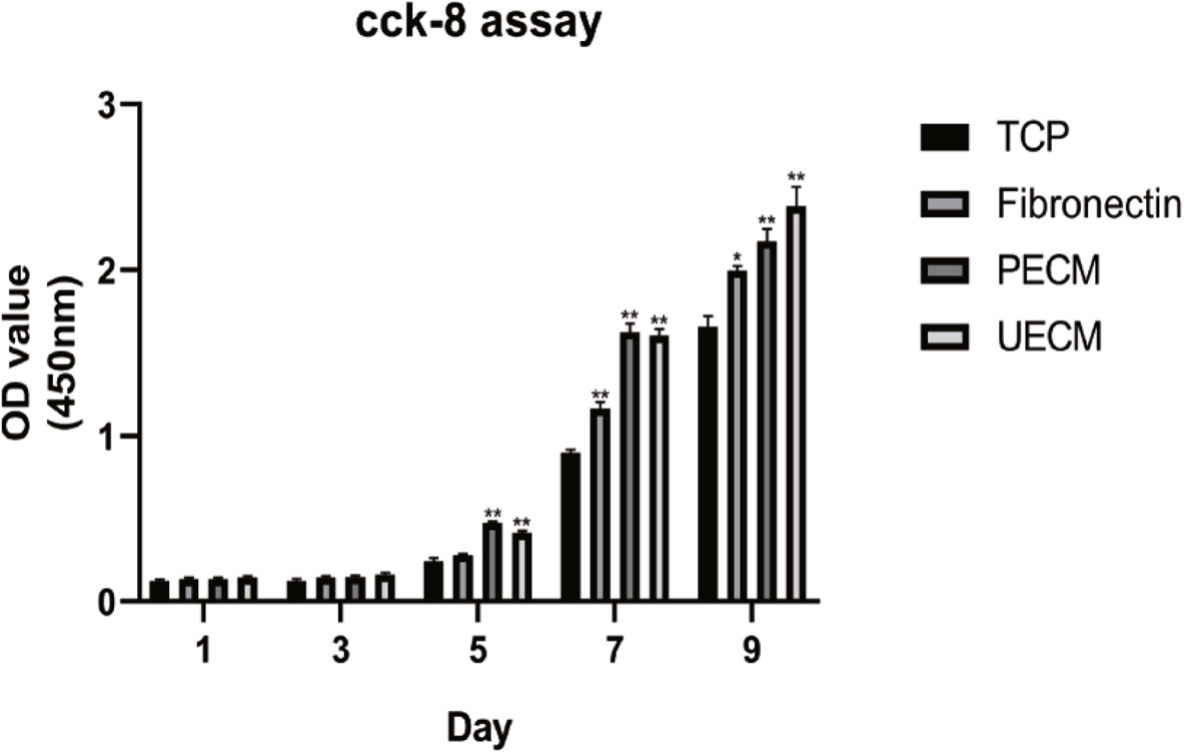
The effects of different substrates on the proliferation of hPDLSCs according to a CCK-8 assay. All values indicate the mean ± SD; *p < 0.05 and **p < 0.01

hPDLSC proliferation was assessed using a CCK-8 assay (CCK-8 kit, Dojindo), which showed that the proliferation of hPDLSCs was significantly elevated when they were grown on UECM and PECM but slightly elevated when they were grown on fibronectin compared to hPDLSCs growth in the blank control group. On day 9, the growth rate of hPDLSCs on UECM was even higher than that on PECM (Fig. 3).

### hPDLSCs adhere to ECM

**Fig. 4:**
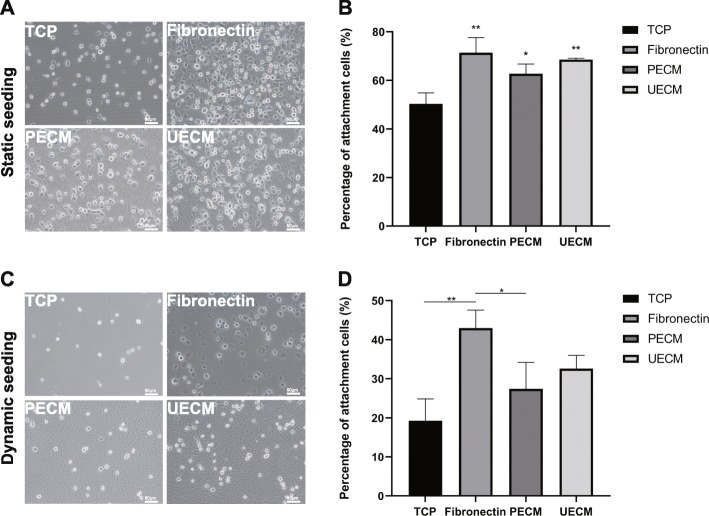
hPDLSC adhesion to different substrates in serum-free medium. **a**, **b** Static conditions. **c**, **d** Dynamic conditions. **a**, **c** At 1 h after seeding, with non-attached cells removed, the remaining cells on different substrates. Scale bar = 80 μm. **b**, **d** The statistical results of cell adhesion rates. All values represent the mean ± SD; *p < 0.05 and *p* < 0.01

Both static and dynamic methods were used to detect the capability of hPDLSCs to attach to 4 surfaces. Under static conditions, the adhesion rate of hPDLSCs to fibronectin-coated TCP was 71.4%, the highest among the 4 groups. The adhesion rates of hPDLSCs to UECM- and PECM-coated TCP were 68.5% and 62.7%, respectively, while only 50% of cells attached to untreated TCP. Compared to adhesion on TCP, adhesion on TCP coated in the 3 substrates was significantly increased (Fig. 4a, b).

Under dynamic conditions, 51.8% and 41.4% of the cells attached to fibronectin- and UECM-coated TCP, which were significantly higher than the percentage of cells attached to TCP alone (only 28%). The adhesion rate of hPDLSCs on PECM-coated TCP was 41.5%, which was not significantly different than the adhesion rate of hPDLSCs to TCPs (Fig. 4c, d).

### hPDLSC morphology depends on ECM

**Fig. 5:**
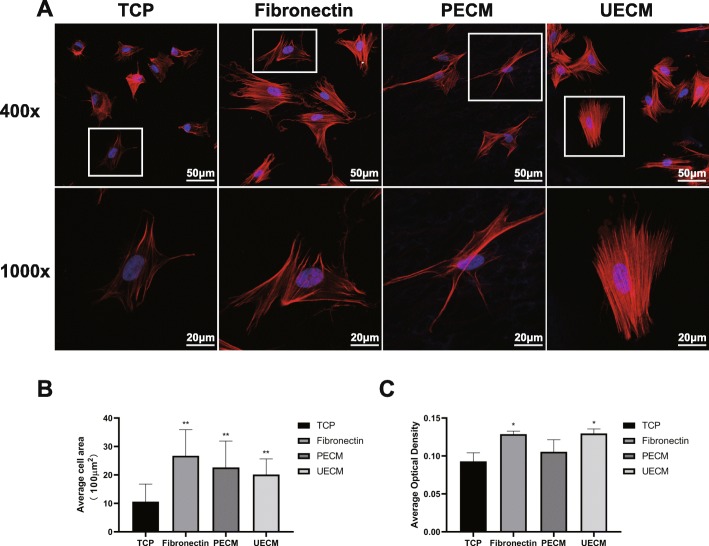
Spreading and morphology of cells grown on ECM. **a** hPDLSC morphologies on different substrates at × 400 and × 1000 magnification. Actin filaments are stained in red by phalloidin, and the nucleus is stained in blue by DAPI. **b** Quantification of cell area and **c** the average optimal density of the fluorescence using NIH Image J. All values are mean ± SD; *p < 0.05 and **p < 0.01

Six hours after seeding, hPDLSCs stained with phalloidin-Atto 555 and DAPI were visualized using confocal laser scanning microscopy. ImageJ software was used to assess the extent of cellular staining. Compared to cells seeded on uncoated TCP, which exhibited a round shape, well-developed actin stress fibers were apparent in the other 3 groups, suggesting increased cell adhesion activity (Fig. 5a). ImageJ analysis showed that the average areas of fibronectin-, PECM-, and UECM-coated TCP to which cells had adhered were approximately 2674.6 μm^2^, 2344.3 μm^2^, and 2135.0 μm^2^, respectively, while only 1165.8 μm^2^ of untreated TCP contained adhered cells. Fibronectin, PECM, and UECM all promoted hPDLSC spreading. However, cells grown on fibronectin exhibited the maximum surface area (Fig. 5b). The average optimal density of the fluorescence showed an upregulation in fibronectin and UECM group (Fig. 5c).

### hPDLSC differentiation on ECM in vitro

hPDLSCs were cultured on 4 different surfaces in osteogenic, adipogenic, and angiogenic media, and their differentiation capacity was assessed.

**Fig. 6:**
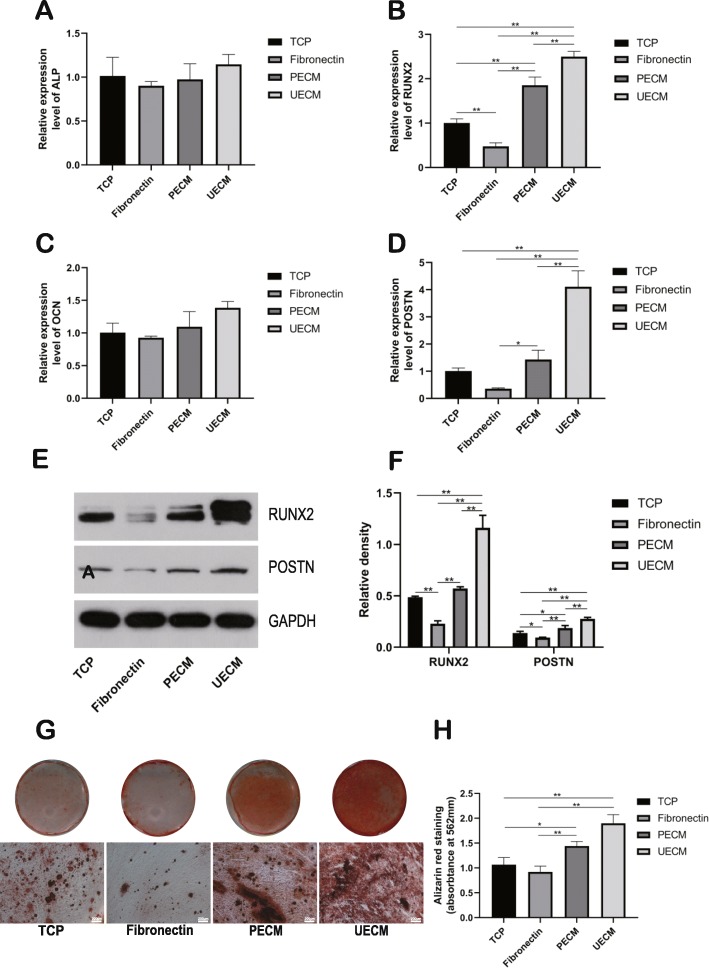
Osteogenic differentiation on different substrates. **a** ALP, **b** RUNX2, **c** OCN, and **d** POSTN expression were analyzed on culture day 7 in osteogenic medium by real-time PCR. **e** RUNX2 and POSTN expression were analyzed on culture day 7 in osteogenic medium by western blot. **f** The relative density of western blot using NIH Image J. **g** After osteogenic induction on different substrates for 21 days, mineral deposits were detected by alizarin red staining. Scale bar = 200 μm. **h** Quantitation of alizarin red staining of day 21 cultures. All values represent the mean ± SD; *p < 0.05 and **p < 0.01

To assess osteogenic differentiation, RT-PCR was conducted on day 7 to detect the gene expression of ALP, RUNX2, OCN, and POSTN. Although hPDLSCs cultured on UECM showed higher expression of ALP and OCN, there was no significant difference among the four groups (Fig. 6a, c). The gene expression of RUNX2 and POSTN were the highest when hPDLSCs were cultured on UECM (Fig. 6b, d). Furthermore, the same trend in protein expression of RUNX2 and POSTN was observed (Fig. 6e, f). After the hPDLSCs were incubated in osteogenic medium for 21 days, alizarin red staining was performed, and the staining intensity was qualified, which indicated that the hPDLSCs cultured on UECM exhibited the strongest level of staining (Fig. 6g, h). Although the staining of fibronectin group was weaker than that of TCP group, there was no significant difference between them according to the qualification of alizarin red staining (Fig. 6g, h). These data showed that hPDLSCs on UECM underwent the highest level of osteogenesis.

**Fig. 7:**
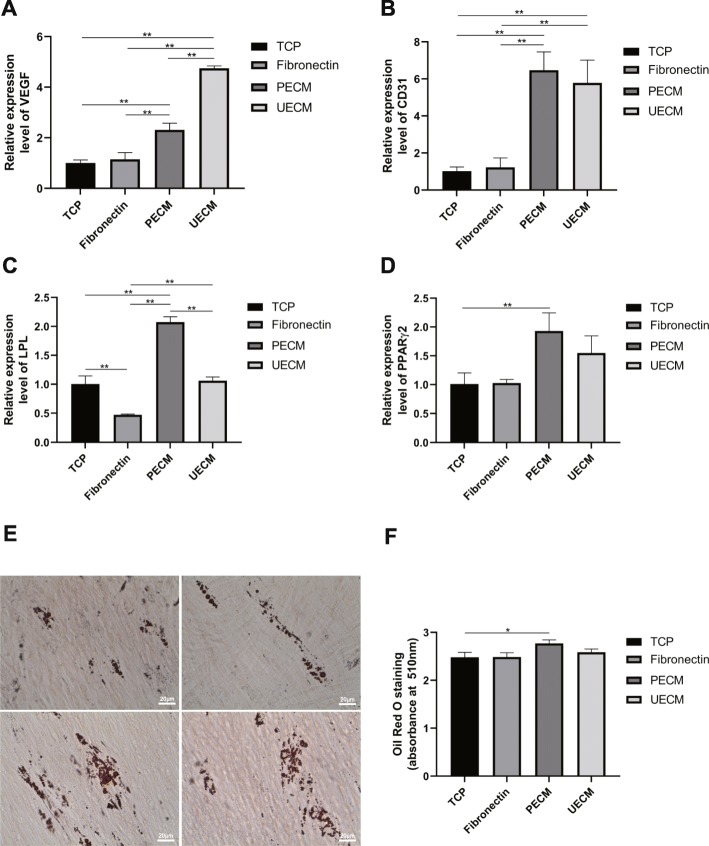
Angiogenic and adipogenic differentiation on different substrates. **a**, **b** Angiogenesis-related genes. **a** VEGF and **b** CD31 expression analyzed on culture day 7 in angiogenic medium by real-time RT-PCR. **c**, **d** Adipogenesis-related genes. **c** LPL and **d** PPARγ2 expression levels were analyzed on culture day 7 in adipogenic medium by real-time RT-PCR. **e** After adipogenic induction on different substrates for 21 days, lipid-laden lobules were detected by Oil Red O staining. Scale bar = 20 μm. **f** Quantitation of Oil Red O staining of day 21 cultures after isopropanol extraction. All values represent the mean ± SD; *p < 0.05 and **p < 0.01

To assess angiogenic differentiation, RT-PCR was performed after hPDLSCs were cultured on angiogenic medium for 7 days. The expression levels of both VEGF and CD31 were upregulated in the UECM and PECM groups (Fig. 7a, b).

To assess adipogenic differentiation, RT-PCR was conducted, which revealed that the adipogenic markers LPL and PPARγ2 were upregulated only in the PECM group (Fig. 7c, d). Oil Red O staining at day 21 was also strongest in the PECM group. The results of RT-PCR and Oil Red O staining both suggest that only the PECM group promote adipogenic differentiation potential of hPDLSCs (Fig. 7e, f).

### Flow cytometry analysis of hPDLSCs on different surfaces

**Fig. 8:**
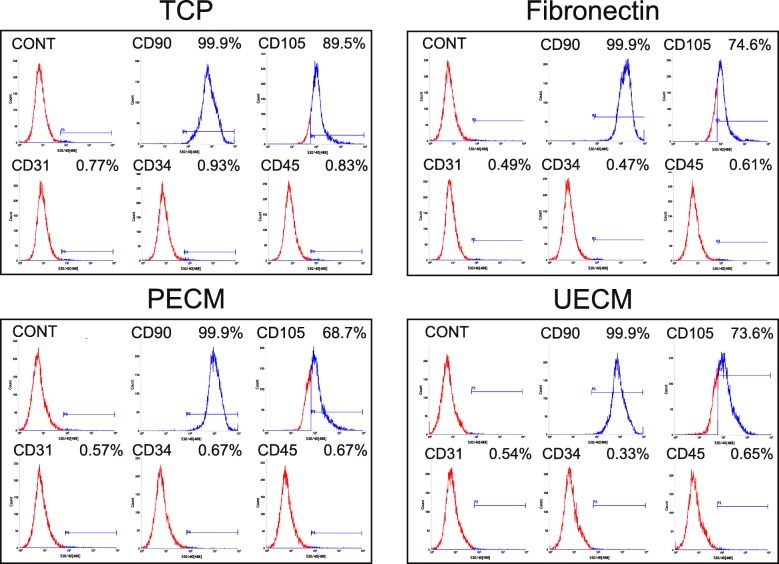
Flow cytometry analysis of hPDLSCs grown on different surfaces

The flow cytometry analysis results showed that hPDLSCs expressed a similar immunophenotype of CD31, CD34, CD45, and CD90 after culturing on TCP, fibronectin, PECM, and UECM for one generation. The positive rates of CD105 were respectively 89.5%, 74.6%, 68.7%, and 73.6% in TCP, fibronectin, PECM, and UECM group, showing a slight difference (Fig. 8).

### hPDLSC differentiation on ECM in vivo

**Fig. 9:**
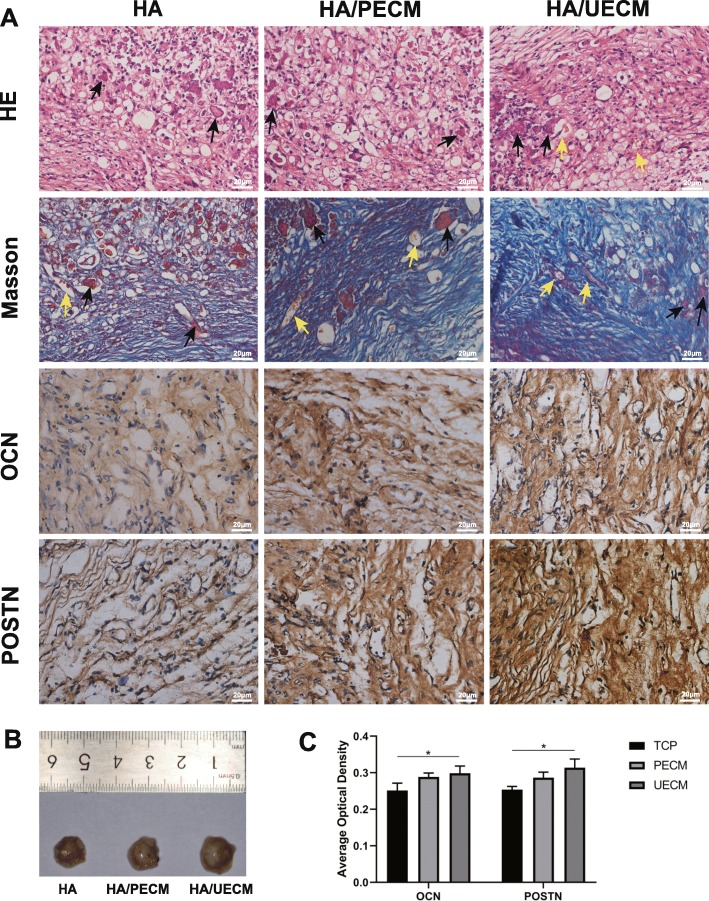
hPDLSC differentiation on ECM in vivo. **a** Analysis of explants at 6 weeks after subcutaneous implantation. Representative images of slides with hematoxylin and eosin staining, Masson’s trichrome staining, and immunohistochemical staining of OCN and POSTN. Representative images of explant HA powder and vessels were noted by black and yellow arrows, respectively. Scale bar = 20 μm. **b** Representative images of explants. **c** The quantification of the average optical density. All values represent the mean ± SD; *p < 0.05 and **p < 0.01

After 6 weeks of implantation, explants were harvested. Tissue infiltration penetrating and vasculature could be observed by nude eyes upon the implanted mixtures (Fig. 9b).

HE staining showed a rich network of fibrous connective tissue in all groups with more vessel formation in the HA/UECM group. More abundant collagen fibers were found in the HA/UECM and HA/PECM groups compared to that in the HA group by Masson staining. Furthermore, immunohistochemical staining showed that the expressions of POSTN and OCN were highest in the HA/UECM, slightly higher than that in the PECM group (Fig. 9a). The quantification of the average optical density showed the same trend (Fig. 9c).

## Discussion

In this study, we investigated the biological influence of ECM derived from hUSCs on the proliferation, adhesion, spreading, and differentiation of hPDLSCs. We used hPDLSCs grown on PECM-coated TCP, fibronectin-coated TCP, and uncoated TCP as control groups and compare changes in hPDLSCs. The present results indicate that UECM can accelerate the proliferation, adhesion, spreading, and osteogenic and angiogenic differentiation of hPDLSCs. UECM in combination with HA powder significantly promoted fibrous connective tissue formation in nude mice compared to that in hPDLSCs loaded with pure HA powder that were cultured on TCP. hPDLSCs cultured on UECM- and PECM-coated TCP had similar growth curves and grew dramatically faster than cells cultured on fibronectin-coated and uncoated TCP. Moreover, UECM promoted osteogenic and angiogenic differentiation to a greater extent than that observed in other groups of hPDLSCs, while PECM promoted the adipogenic differentiation of hPDLSCs. Fibronectin had the most positive effect on hPDLSC adhesion and spreading, but did not promote osteogenic differentiation. Fibronectin, an abundant component of UECM, exhibits biological behaviors different from those of UECM. Stem cells are typically cultured on TCP, on which they lose many characteristics. ECM is a promising candidate to increase stem cell expansion and enhance the biological capabilities of stem cells, such as differentiation, spreading, and attachment. Previous findings strongly indicate that MSC-derived ECM has great potential as a bioactive scaffold. However, studies on MSC-derived ECM have mainly focused on BMSCs, while studies on ECM derived from other MSCs are rare. As one of the most promising cell sources for complex periodontal tissue, hPDLSCs have been examined in many in vitro and in vivo experiments and even in clinical trials [31]. It is important to find a suitable substrate for periodontal regeneration that can expand hPDLSCs on a large scale and increase their biological functions. hUSCs, which are isolated from human urine, exhibit the characteristics of MSCs including self-renewal and multipotential differentiation, and are applied in renal reconstruction/kidney bioengineering, genitourinary repair, neurodegeneration, cardiac repair, liver reconstruction, and bone engineering [26, 27, 32, 33]. hUSCs have several advantages over other MSCs; these include their non-invasive collection method, low cost, and safety.

Though Pei et al. have studied the effect of ECM deposited by non-chondrogenic hUSCs on hBMSCs and shown that UECM enhances the expansion and chondrogenic capacity of hBMSCs after repeated passage, more attention should be paid to UECM. Furthermore, in Pei et al.’s experiment, a line of hUSCs that does not completely represent primary cells isolated directly from people was used [28]. Heng et al. and Hamano et al. detected the influence of PECM on induced pluripotent stem (iPS) cells and human dental pulp stem cells (DPSCs) and demonstrated that PECM increases proliferation and multipotency [34, 35]. However, few studies have focused on the use of PECM to culture hPDLSCs.

In this study, we used primary hUSCs instead of an hUSC line to produce ECM and compared the differences between the native ECM of hPDLSCs and that of UECM. Both ECMs consisted of dense bundles of fibers with more small inter-holes and curly fibers found in UECM than in PECM. ECMs vary in their concentration and composition, leading to different properties. We analyzed UECM with immunofluorescent staining and found that fibronectin is more abundant than collagen I in UECM, while laminin 5 is rare. However, laminins are structural components of the basal lamina that are widespread in the fibrous networks of most cells and organs, where they play a key role in cell adhesion, proliferation, differentiation, and migration [36, 37].

We also used hPDLSCs grown on TCP covered in fibronectin, an abundant component in UECM, as a control group. In the proliferation assay, cells cultured on UECM- and PECM-coated TCP had similar growth rates and grew much faster than the fibronectin and TCP groups. Cells grew faster on fibronectin than those in the TCP group, indicating that individual components of ECM proteins, such as fibronectin, improve the proliferation of stem cells to a degree, but there are still gaps between natural ECM. Previous studies demonstrated that fibronectin does not significantly impact the differentiation of MSCs [38]. Although our results indicate that though fibronectin inhibits expression of RUNX2 and POSTN, there was no significant difference in qualification of alizarin red staining compared to TCP group. Fibronectin appears to be an effective substrate to promote hPDLSC spreading and attaching, but it is not the major factors of UECM in promoting biological behavior of hPDLSCs. However, the key bioactive components that regulate stem cell fate remain unidentified. The previous researches mainly concentrated on the factors such as surface adhesion receptors, multiple protein domains which can bind and release corresponding growth factors, stiffness, physical topography, and deformability of ECM. Rarely proteomic studies have been performed on ECM [13, 19–22]. Lin et al. have assessed components of hBMSC-derived ECM finding there is a complex mixture of different protein components [15]. The concentration of fibronectin is likely one of the factors that influences the biological characteristics of ECM. Our group is attempting to perform a precise qualification of proteins in UECM in our next study.

Previous findings have strongly suggested that MSC-derived ECM is a good bioactive scaffold that can maintain stemness, rejuvenate aged stem cells, and enhance the differentiation potential of stem cells [12, 14–18]. We assessed the immunophenotype of hPDLSCs after culturing on TCP, fibronectin, PECM, and UECM for one generation. No significant difference was found among the 4 groups except the expression of CD105 which was slightly higher in TCP group. However, a more significant difference may appear with prolonged culture time. We will further examine how UECM contributes to the characteristics of MSCs in our next study.

Concerning osteogenic differentiation, UECM upregulated the gene and protein expression of RUNX2 and POSTN. POSTN, a 90-kDa ECM protein, is expressed at high levels in the periosteum, periodontal tissues, bones, tendons, and skins, where it plays a crucial role in bone formation and metabolism [39–41]. Zhang et al. found that POSTN promotes the osteogenic differentiation of MSCs possibly via the Wnt signaling pathway [42]. Wu et al. studied interactions between POSTN and hPDLSCs and showed that POSTN enhances the migration, proliferation, and differentiation of hPDLSCs [43]. Furthermore, Tang et al. discovered that POSTN promotes the migration and osteogenic differentiation of hPDLSCs under inflammatory conditions via the Jun amino-terminal kinases (JNK) pathway. The domains of POSTN can bind to Notch1 and CCN3 to maintain cell stemness [41]. Previous findings strongly suggest that POSTN is an important protein that affects the morphogenesis and development of bone and periodontal tissues [44]. Perhaps, UECM promotes differentiation of hPDLSCs by upregulating the expression of POSTN, which plays a pivotal role in various biological functions, in hPDLSCs. As a specific pro-angiogenic factor, VEGF plays an important role in the overall process of angiogenesis. The expression of VEGF can be observed in many normal tissues, but it is increased in angiogenic tissues [45]. Many factors can affect the expression of VEGF, such as hypoxia, which can significantly promote the synthesis of VEGF. In periodontal tissue regeneration, ensuring an abundant blood supply in the defect area is an important step [46]. UECM can promote the expression of VEGF in hPDLSCs, which has the potential to accelerate revascularization in periodontal defects. UECM also promotes the expression of CD31, suggesting that UECM can not only enhance the angiogenic differentiation of hPDLSCs, but also promote angiogenesis by promoting the expression of VEGF in hPDLSCs.

There was an obvious difference in cell morphology and the extent to which cells spread on different surfaces. Cells grown on PECM exhibited a shuttle-shaped morphology with stretched actin, while cells grown on UECM and fibronectin spread and were shaped like polygons. The extent to which cells spread depends on matrix stiffness, matrix geometry, initial adhesive position, ligand presentation, and ligand density on the surface [12, 47]. The spreading of hPDLSCs into different shapes probably indicates different microstructures present in UECM, PECM, and fibronectin. As shown by the adhesion assay, fibronectin bound to hPDLSCs with the highest affinity and bound better than UECM and PECM at 1 h after cell seeding under both static and dynamic conditions. The same phenomenon was observed in an experiment by Decaris and Leach [14].

ECM can regulate intracellular signaling via cell surface adhesion receptors. Lysophosphatidic acid (LPA), as a bioactive phospholipid that is widely expressed in ECM, participates in the regulation of multiple biological behaviors of cells, such as cell adhesion, proliferation, migration, and differentiation, by activating G-coupled LPA receptors. LPA has a positive effect on osteogenic differentiation of human MSCs [48]. Additionally, LPA has been reported to enhance differentiation of erythrocytes and promote vessel formation in vertebrates [49, 50]. It can also stimulate human trabecular meshwork (TM) cells to produce additional ECM by activating the YAP/TAZ transcriptional pathway [51]. Nevertheless, LPA negatively regulates adipogenesis [52–54]. In our experiment, LPA likely contributed to the series of biological process in hPDLSCs by activating its receptors leading the differences among 4 groups. We are interested in the connection between LPA and ECM.

Advantages of UECM were confirmed by in vivo heterotopic regeneration experiments in nude mice. More abundant collagens were observed in the UECM/HA group and PECM/HA group with the highest expression of OCN and POSTN in the UECM/HA group, which play an essential role in periodontal regeneration. Compared to previous methods which only mixed seed cells with HA powder [18], we mixed ECMs with HA powder before loading cells to make ECMs work in vivo sequentially.

The limitations of this research derive from a lack of precise quantification of the protein concentration of ECM. Identical numbers of cells were stimulated for the same amount of time (8 days) to produce ECMs. However, the final concentration of ECM was difficult to measure because the process that produces ECM is affected by various factors.

In our experiment, both UECM and PECM markedly accelerated hPDLSC proliferation. However, UECM was more advantageous for the osteogenesis, angiogenesis, and adhesion of hPDLSCs than PECM. Fibronectin promoted adhesion and spreading of hPDLSCs to the greatest extent, but did not promote osteogenesis. hUSCs are attractive because they are easily harvested, non-invasive, and low in cost, making the industrial production of ECM possible. As ECM is a desirable biological scaffold, it can also be coated onto or incorporated into synthetic biomaterials [55]. Recently, decellularized ECM bioink has gained extensive attention as it can be utilized in tissue engineering, drug screening, and in vitro disease models. The production of decellularized ECM bioink involves first dissolving the ECM, then printing cell-laden decellularized ECM gels with a polymeric framework, followed by fabricating 3D structures layer by layer [56]. UECM with the advantages mentioned above may be a promising agent for use in 3D bioink projects or biological scaffolds. However, additional biological assays to determine the mechanism of the interaction between ECM and hPDLSCs are worthy of deeper exploration.

## Conclusion

In summary, UECM consists of dense bundles of fibers which contain abundant fibronectin and our study provides an original perspective on the use of ECM specific to different cell types. UECM promoted hPDLSC proliferation, spreading, adhesion, and osteogenic and angiogenic differentiation and exhibited advantages over PECM and fibronectin. UECM is a promising biomaterial for periodontal tissue regeneration that deserves more attention and requires deeper exploration.

## Supplementary information

**Additional file 1.** Conformation for the primers working. The DNA gel shows a good specificity of the primers including ALP, RUNX2, OCN, POSTN, VEGF-A, CD31, LPL, PPARγ2, GADPH. The file is for review purpose only.

**Additional file 2.** Spreading and morphology of cells grown on ECM. in 3D representation. Cells cultured on 4 surfaces were stained with phalloidin-Atto 555 and DAPI and were scanned for 3D layer by confocal laser scanning microscopy. Cells represent more like 2D morphologies. The file is for review purpose only.

**Additional file 3 : Figure S1.** The file is for review purpose only.

**Additional file 4 : Figure S2.** The file is for review purpose only.

## Data Availability

Not applicable.

## Abbreviations

ECM: Extracellular matrix
EDTA: Ethylenediaminetetraacetic acid
FBS: Fetal bovine serum
H&E: Hematoxylin and eosin
hPDLSCs: Human periodontal ligament stem cells
hUSCs: Human urine-derived stem cells
MSCs: Mesenchymal stem cells
OCN: Osteocalcin
PECM: ECM derived from hPDLSCs
POSTN: Periostin
PVDF: Polyvinylidene fluoride
RT-PCR: Real-time polymerase chain reaction
RUNX2: Runt-related gene 2
SDS-PAGE: Sodium dodecyl sulfate polyacrylamide gel electrophoresis
SEM: Scanning electron microscopy
TCP: Tissue culture plastic
UECM: ECM derived from hUSCs
WB: Western blot
α-MEM: α-Minimum essential medium
