# Supplementary figures and images for: Extracellular matrix derived from human urine-derived stem cells enhances the expansion, adhesion, spreading, and differentiation of human periodontal ligament stem cells
Source: Stem Cell Res Ther. 2019 Dec 18;10:396. doi: 10.1186/s13287-019-1483-7 (PMC6921428; doi:10.1186/s13287-019-1483-7)

GAPDH PPAR $\gamma$  LPL CD31 VEGF POSTN OCN RUNX2 ALP

50bp

300bp

600bp

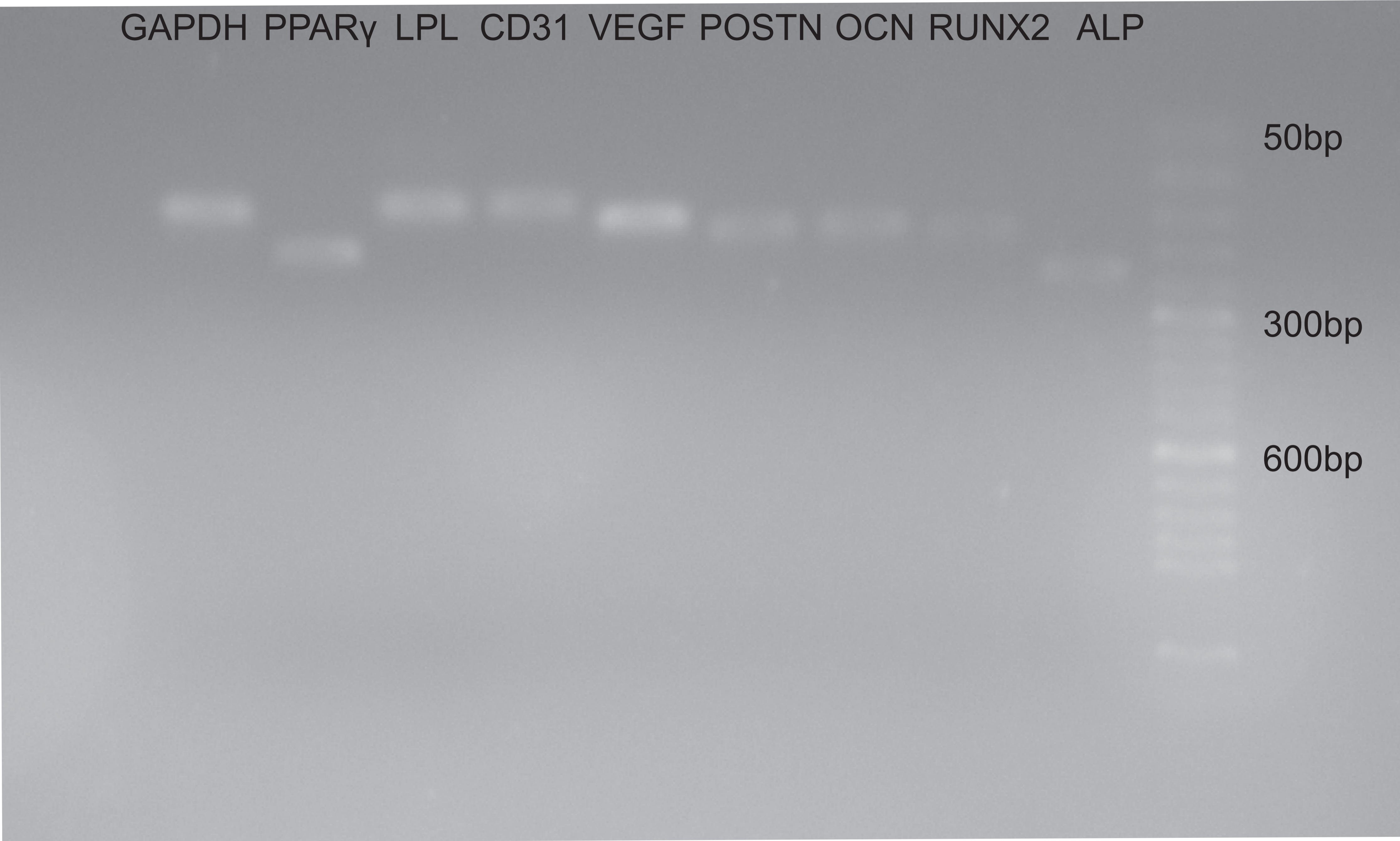

Supplement: Supplementary file 1 — Additional file 1. Conformation for the primers working. The DNA gel shows a good specificity of the primers including ALP, RUNX2, OCN, POSTN, VEGF-A, CD31, LPL, PPARγ2, GADPH. The file is for review purpose only. [file 13287_2019_1483_MOESM1_ESM.pdf]

TCP

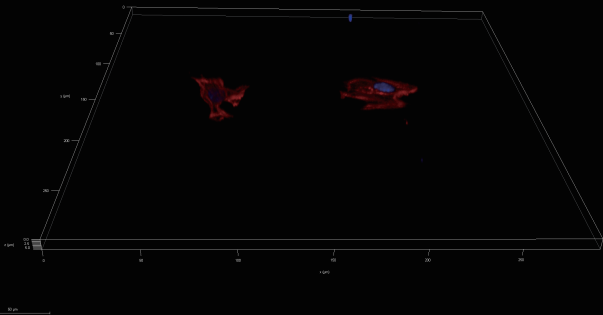

Fibronectin

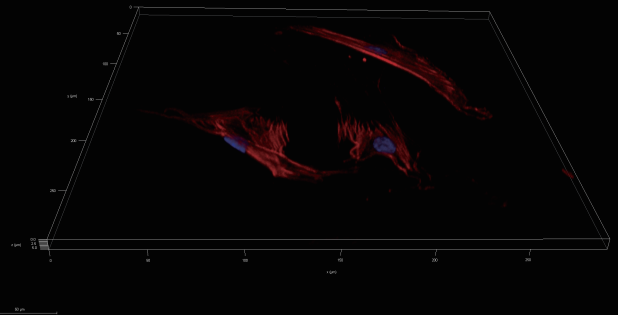

PECM

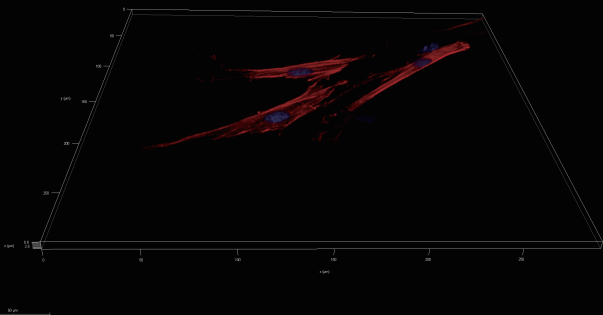

UECM

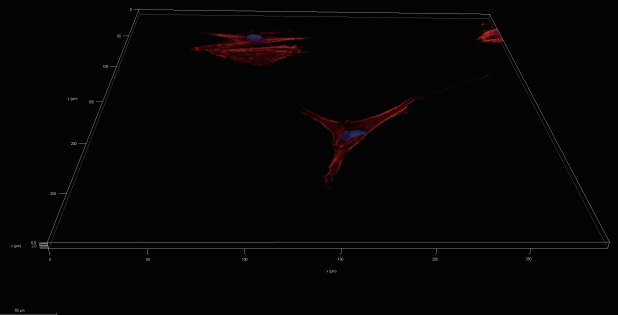

Supplement: Supplementary file 2 — Additional file 2. Spreading and morphology of cells grown on ECM. in 3D representation. Cells cultured on 4 surfaces were stained with phalloidin-Atto 555 and DAPI and were scanned for 3D layer by confocal laser scanning microscopy. Cells represent more like 2D morphologies. The file is for review purpose only. [file 13287_2019_1483_MOESM2_ESM.pdf]

**A**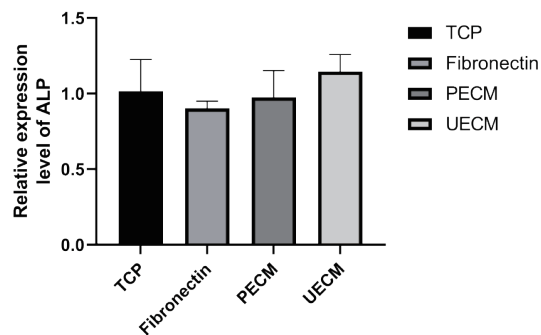**B**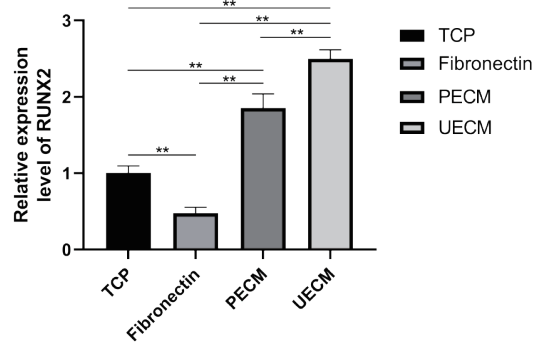**C**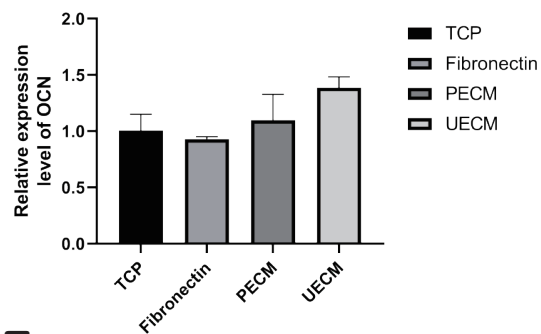**D**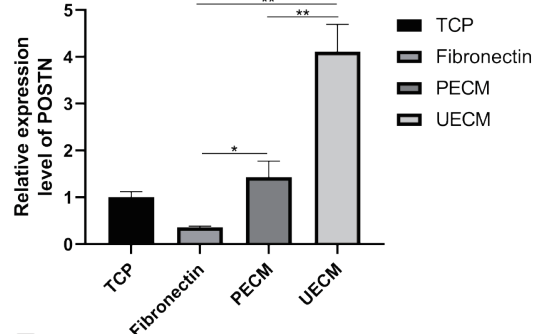**E**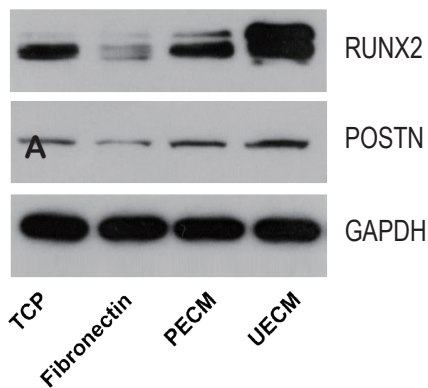**F**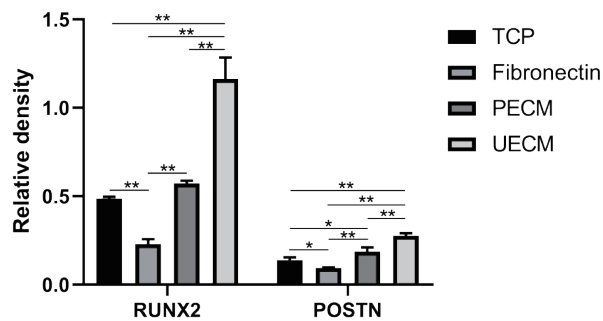**G**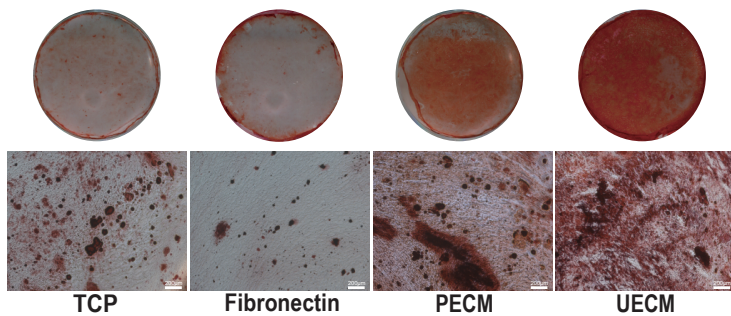**H**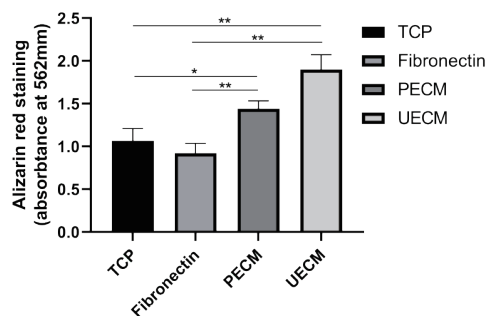

Supplement: Supplementary file 4 — Additional file 4 : Figure S2. The file is for review purpose only. [file 13287_2019_1483_MOESM4_ESM.pdf]
