# Supplementary material for: Extracellular matrix derived from human urine-derived stem cells enhances the expansion, adhesion, spreading, and differentiation of human periodontal ligament stem cells
Source: Stem Cell Res Ther. 2019 Dec 18;10:396. doi: 10.1186/s13287-019-1483-7 (PMC6921428; doi:10.1186/s13287-019-1483-7)
Supplement: Supplementary file 3 — Additional file 3 : Figure S1. The file is for review purpose only. [file 13287_2019_1483_MOESM3_ESM.pdf]

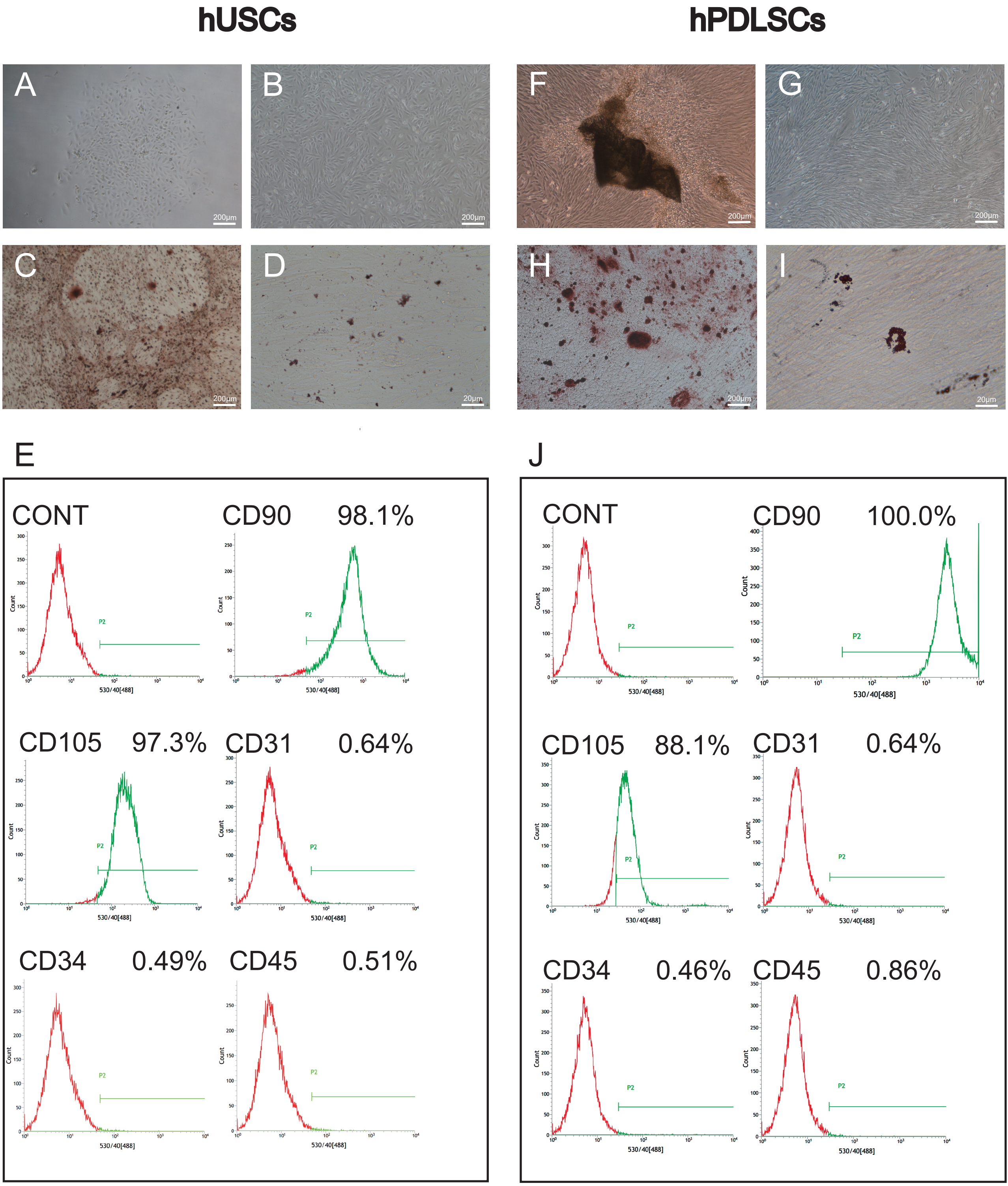
hUSCs
hPDLSCs
A
B
C
D
F
G
H
I
200μm
20μm
E
J
CONT
CD90 98.1%
CD105 97.3%
CD31 0.64%
CD34 0.49%
CD45 0.51%
CONT
CD90 100.0%
CD105 88.1%
CD31 0.64%
CD34 0.46%
CD45 0.86%
Count
530/40[488]
P2

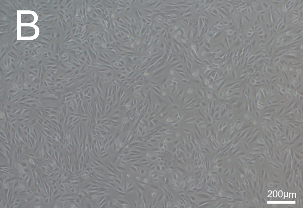
B
200μm

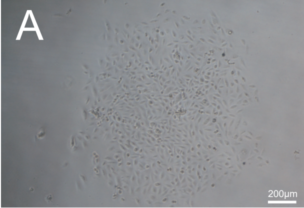
A
200μm

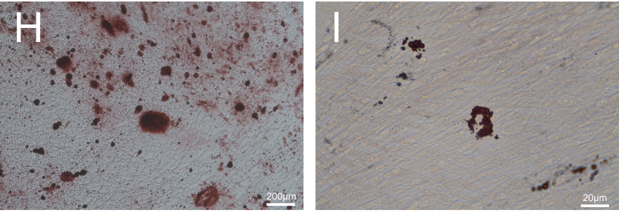
H
200µm
I
20µm
